# Commentary: Automatic chemistry and the human protein index

**DOI:** 10.1155/S1463924680000521

**Published:** 1980


### Commentary

### Automatic chemistry and the human protein index

It is now technically feasible to assemble a nearly complete index of all of the proteins (or protein subunits) of man [1-3]. It is estimated that the human genome contains between 30,000 and 50,000 structural genes, and that perhaps 10% of these genes are active in any given cell type. Hence the analytical systems required for this effort must be able to analyse quantitatively mixtures of 3,000 to 5,000 proteins to deal effectively with single cell types, and ultimately to resolve the entire set of up to 50,000 if the index is to be properly constructed. Present two-dimensional electrophoretic systems resolve well over 2,000 proteins or protein subunits, but do not represent as yet the limits of resolution that can be reached. Fortunately sample requirements are very small, being in the order of 1 mg of protein for Coomassie blue stained gels, 10$\mu$g of protein for silver staining, and, depending on the amount of label incorporated and its specific activity, well below the nanogram range for autoradiography or fluorography.

It is apparent to many workers in this field that a revolution in medicine could occur if the thousands of spots seen can be identified and quantitated on replicate gels with precision, if new efficient methods could be developed for identifying known enzymes and antigens in the patterns, if the analyses could be done quickly and inexpensively in the clinical laboratory setting, and if the data could be analysed efficiently using small dedicated computing systems.

The types of problems which could be attacked are legion, and initially one would attempt survey studies to find useful disease correlations, as has been done with nearly all new assays in the past. If it is worthwhile to examine the alterations in the level of one protein such as alpha-feto-protein in plasma in a wide variety of patients for example, then it follows that it would be much better to be able simultaneously to examine quantitatively 2,000 proteins in the same series of diseases. However, this requires some readjustment in perspective.

The first readjustment comes from the realisation that most proteins are unknown, unnamed, and have unknown functions. Protein isolation usually follows from the discovery of a function or activity, and possibly a thousand human enzymes and other proteins (2-3% of the total) have been characterised to some degree, and a lesser number isolated in pure form.

The second readjustment concerns the relationship of specific protein analysis, especially of tissue proteins, to clinical chemistry. The bulk of the tests now done indicate changes in amount of a substance, usually of low molecular weight. Two-dimensional protein maps in contrast, *may* indicate the fundamental lesion in the form of an altered or absent protein, i.e. may identify the cause.

The third, and in some respects, most difficult, readjustment relates to the way research in disease processes may be conducted. Some present animal research may no longer be needed or be relevant. This follows from the thought that most human disease is ultimately to be understood in terms of alterations in the structure, amount, location, or time of appearance during development of specific proteins or protein sets. We do not believe it will be feasible to map or index animal cells and tissues as completely as will be done for human samples, largely because of the cost, time, and effort involved. An exception may be primates which closely resemble man, and which may be used to study changes in proteins during various stages of development where human material is difficult to obtain. Confirmatory studies on human tissues would ultimately be required, however. To be relevant, an animal model must be shown to be identical or similar to a human disease in terms of the specific proteins involved. To do this means in many cases, that the human disease must be largely understood. Note in addition that one reason for using animal tissues has been that sufficient human material was not available. The extraordinarily small samples required for 2-D electrophoresies remove this problem.

### The need for automation

While over 30,000 two-dimensional electrophoretic analyses have already been run in this laboratory [4-5], the automation of the entire system is in a very primitive stage. We believe that automation is essential to lower cost, and to obtain reproducibility. Not only the electrophoretic step, but the subsequent image analysis and computerised data reduction must be made simple, automatic, and as far as possible, miniaturised. That, however, is far from the end of the problem. The aim is to be able to identify *each* protein uniquely, to be able to distinguish it from all others found in human cells, and to also establish identities, i.e. that a spot seen in a 2-D brain protein gel is identical to one seen in a kidney protein gel; or that a new protein found in a tumor cell is really the same as one found at some stage in embryogenisis. To achieve these goals, it is necessary to be able to run very large numbers of analyses indeed.

This requirement follows from the fact that samples must be run at several different loadings to achieve maximum resolution, and that additional runs must be made using internal standards for pI [6] and molecular weight [7]. Identity of spots is established in a variety of ways, including comigration, similar amino acid analysis (obtained by analysing a number of samples of the same cell labelled with different amino acids), formation of similar carbamylation trains, and by showing that the thermal denaturation curves are similar. In addition single spots may be partially digested and shown to give similar patterns in so-called Cleveland gels [8].

Ultimately, however, two difficult problems must be solved. The first is the identification of spots corresponding to all known enzymes. We have done sufficient protein isolation using classical methods to conclude that some other systematic approach is required. Use of thermal denaturation techniques has been explored, and offers promise [9], but does not appear to be the final solution.

The second problem is that of definitive localisation of a given protein in one cell type found in a mixed tissue. Cell separation is of assistance, but may not give clean cut results. In addition, when the appearance of a protein or a set of coregulated proteins appears during differentiation, especially in the very early embryo, sufficient material may not be available, and subtle difference may exist between cells which are morphologically identical.

The obvious answer to the problem is to make antibodies, preferably monoclonal antibodies, against as many different proteins or protein subunits as possible. The large amount of work required to sort out useful antibody cells from hybridoma mixtures also calls for automation on a scale not previously envisioned.

### Pattern of biomedical research

It might be thought that an effort to produce a human protein index, and to extend it with specific reagents (antibodies) for each entry, would be an impossibly large undertaking, difficult to manage and organise. The truth is that not only is it difficult, it is impossible given the traditional *modus operandi* of biomedical research. It would not be possible, for example, to launch a truly interdisciplinary project such as this one and depend on a grant system in which each part is separately reviewed and funded, and where as much as a year's delay may occur between the identification of a need, and funding to provide a solution. It is also evident that the problem cannot be solved simply by accretion, i.e., by hoping to compile the index from uncorrelated studies in many different laboratories. High-energy physics mastered the art of organising and running large collaborative ventures. In contrast, the biomedical sciences have so far avoided them, possibly because they were not really needed.

Now, however, we face, really for the first time, the true complexity of the human cellular systems with which we work. Extraordinary reluctance to alter the pattern or scale of biomedical research is to be expected. It is important to inquire briefly, therefore, into what could be gained if an intensive effort to produce a human protein index were made, and what would be the alternative.

Of central importance are the annotations which will accompany each numbered entry in the human protein index. These would include (following the gel spot number):

1. The sample source.
2. Molecular weight (actual position on an SDS gel).
3. Isoelectric point.
4. Name or function where known.
5. Subcellular localisation of the protein.
6. Associated subunits if a multimeric protein.
7. Identification of the coregulational set to which a protein belongs.
8. Chromosomal location of the structural gene for the protein or protein subunit.
9. Variants of a peptide and their correlation with disease.
10. Spot co-ordinates on a standardised reference map.
11. Notes including all observations relative to a peptide including response to experimental variable.
12. Corresponding master human protein index number.

We describe elsewhere the rules and rationale for initial number of spots on gels, and we propose to delay for some time the assignment of master numbers. Obviously the index can be searched from the viewpoint of any class of entry. One could, for example, obtain a list of all of the proteins known to be coded for by genes on chromosome I.

For the purposes of this discussion the important points are that proteins and protein sets characteristic of discrete cell types, of stages in development, of germ layers, and very probably of a number of disease states will be discoverable by searching through the index data base [10]. Of special interest is the problem of corregulated sets. Are genes expressed, (for this discussion lumping together all stages and processes between DNA and protein) in sets or batteries, and are these sets expressed in a precise order during development? In cancer, are sets characteristic of early developmental stages turned back on? These are questions which cannot be answered without the mass of analytical data necessary to form the index.

The alternative is to slowly explore human cells by classical techniques, isolating one protein after another, and exploring its relation to disease in a laborious fashion. As correlations with disease are discovered, new immunoassays can then be developed. Given tens of thousands of proteins to explore, and an almost equal number of diseases to correlate each one with, such an approach will be time consuming and ultimately very expensive indeed.

The most compelling argument for the more global approach offered by analytical systems which 'see' a very large number of discrete entities at a time comes from simple statistical considerations. If one causal anomalous protein is to be discovered in a set of 30,000 for example, there is a 1% chance that it will be among the first 1% examined by classical methods, and there is an equal chance that it will be among the *last* 1% studied.

Further, the prospect of analysing samples from patients with thousands of different diseases (over 2,000 human genetic diseases are known) one protein at a time is dismal indeed.

We rather tend to the view that a well planned and integrated attempt to develop automatic systems for sample preparation, for two-dimensional electrophoresis, for spot identification, for image analysis and data reduction, for hybridoma cell selection, and for computerised search programs to find correlations between patterns or variants and disease would in the end contribute not only to more precise diagnosis, but to the evaluation of therapy, and to the discovery of new protein replacement therapies.

*Norman G. Anderson and Leigh Anderson*

REFERENCES

[1] O Farrell, P.H. *J. Biol.Chem.*, 1975, **250**, 4007.
[2] Anderson, N.G., and Anderson, N.L., *Behring Inst. Mitt*, 1979, **63**, 169.
[3] Anderson, N.L., Edwards, J.J., Giometti, C.S., Willard, K.E., Tollaksen, S.L., Nance, S.L., Hickman, B.J., Taylor, J., Coulter, B., Scandora, A., and Anderson, N.G. in 'Electrophoresis '79', Ed. B.J. Radola. 1980 W. de Gruyter, Berlin. pp. 313-328.
[4] Anderson, N.G., and Anderson, N.L. *Anal. Biochem.* 1978 **85**, 331.
[5] Anderson, N.L., and Anderson, N.G., *Anal. Biochem.* 1978 **85**, 341.
[6] Anderson, N.L., and Hickman, B.J. *Anal. Biochem.* 1979 **93**, 312.
[7] Giometti, C.S., Anderson, N.G., Tollaksen, S.L., Edwards J.J., and Anderson, N.L. *Anal. Biochem.*, 1980, **102**, 47.
[8] Cleveland, D.W., Fishers, S.G., Kirschner, M.W., and Laemmli, U.K. *J. Biol. Chem.*, 1977, **252**, 1102.
[9] Nance, S.L., Hickman, B.J., and Anderson N.L. 'Electrophoresis '79', Ed. Radola, B.J. 1980, W. de Gruyter, Berlin, pp. 351-360.
[10] Taylor, J., Anderson, N.L., Coulter, B.P., Scandora, A.E., and Anderson, N.G. Estimation of two-dimensional spot intensities and positions by modelling. 'Electrophoresis '79', *Ed.* Radola B.J., W. de Gruyter, Berlin 329-339.

